# Assessment of the effects of storage temperature on fatty acid analysis using dried blood spot cards from managed southern white rhinoceroses (*Ceratotherium simum simum*): implications for field collection and nutritional care

**DOI:** 10.7717/peerj.12896

**Published:** 2022-02-14

**Authors:** Jordan Wood, Larry J. Minter, Doug Bibus, Troy N. Tollefson, Kimberly Ange-van Heugten

**Affiliations:** 1 Animal Science, North Carolina State University, Raleigh, North Carolina, United States; 2 North Carolina Zoo, Asheboro, North Carolina, United States; 3 Lipid Technologies LLC, Austin, Minnesota, United States; 4 Mazuri® Exotic Animal Nutrition, PMI Nutrition, Land O’ Lakes, Inc., St. Louis, Missouri, United States

**Keywords:** Dried blood spot, Fatty acid, Rhinoceros, Temperature storage

## Abstract

**Background:**

Southern white rhinoceroses (*Ceratotherium simum simum*) are an endangered species in decline due to poaching and negative habitat changes. Conservation of the species has become increasingly important and a focus on better human management has become prevalent. One area of management that impacts southern white rhinoceroses is nutritional health monitoring, which is often conducted through blood analysis. Blood analysis conducted during field research can be difficult due to temperature, distance, and limited technological resources, so new methods of fast, and relatively stable blood collection are being pursued. One method that has been used in humans for many years is beginning to make its way into wildlife studies: the use of dried blood spot (DBS) cards. These cards are used as a tool to store single drops of whole blood on specialized filter paper and, once dried, can be used for nutritional biomarker analysis. An area of interest for southern white rhinoceroses and nutrition is monitoring fatty acid percentages for cardiovascular, immune, and reproductive health. The time and temperature limitations for storing blood fractions or liquid whole blood when analyzing fatty acids have been investigated, but few studies have performed storage studies on DBS cards colder than −20 °C or in non-human species.

**Methods:**

In order to better understand the limitations of DBS cards and the impact of temperature on fatty acid DBS samples in long-term storage, triplicate samples from seven adult southern white rhinoceroses at the North Carolina Zoo were collected and subjected to three storage treatments (immediate, room temperature (23 °C), or frozen (−80 °C) for 1 year).

**Results:**

Stearidonic (18:4w3) (Δ 0.3%), arachdic (20:0) (Δ 0.1%), eicosatetraenoic (20:4w3) (Δ 0.2%), and erucic acid (22:1w9) (Δ 0.1%) were in higher concentration in frozen than initial. Fatty acids in higher concentrations in the initial samples than frozen were myristic (14:0) (Δ 0.2%), mead (20:3w9) (Δ 0.1%), docosatetraenoic (22:4w6) (Δ 0.2%), nervonic (24:1) (Δ 0.1%), and total highly unsaturated fatty acids (HUFAs) (Δ 0.7%). Stearic (18:0) (Δ 2.2%), stearidonic (18:4w3) (Δ 0.3%), arachdic (20:0) (Δ 0.2%), paullinic (20:1w7) (Δ 0.4%), eicosatetraenoic (20:4w3) (Δ 0.1%), eicosapentaenoic (20:5w3) (Δ 0.1%), docosatetraenoic (22:4w6) (Δ 0.2%), nervonic acid (24:1) (Δ 0.2%), monoenes (Δ 1.9%), and total saturates (Δ 3.6%) had higher concentrations in room temperature than initial. Linoleic (18:2w6) (Δ 4.9%), mead acid (20:3w9) (Δ 0.1%), total polyunsaturated fatty acids (5.3%), and total omega-6 fatty acids (Δ 4.8%) had higher concentrations in initial compared to room temperature. Arachidonic (20:4w6) (Δ 0.4%) and omega-3 docosapentaenoic acid (22:5w3) (Δ 0.1%), had higher concentrations in frozen than in room temperature.

**Discussion:**

The frozen samples had the fewest statistical differences compared to room temperature samples and essential omega-3 and -6 fatty acids were stable with freezing up to 1 year. While more research is still warranted, current results suggest that DBS samples are best utilized when immediate analysis or −80 °C storage is available.

## Introduction

Populations of southern white rhinoceroses (*Ceratotherium simum simum*) have been in decline due to poaching, drought and other negative environmental factors (Emslie, 2020). This has led to discussion of their International Union for Conservation of Nature status being altered to endangered to match the Convention on International Trade in Endangered Species status despite the numbers of white rhinoceroses showing minor increases (Emslie, 2020). Various programs have been enacted to reduce poaching, especially in South Africa where a majority of wild southern white rhinoceroses are found (Dipo, Azizah & Handari, 2020; Ferreira et al., 2017; Knight et al., 2015). However, conservation does not stop at monitoring population numbers; it also requires further understanding of how to maintain species in both free-ranging and managed situations. Field research for nutritional health involves monitoring the health status by using internal and/or external biomarkers such as blood, feces, saliva, and hair (Festa-Bianchet et al., 2017). Field researchers often have to use the least invasive and simplest means of field collection possible, especially in locations like African savannahs and reserves, where technological and biomedical resources are often limited for quick collection and stable storage or processing (Festa-Bianchet et al., 2017).

Proper fatty acid status is important for a variety of physiological systems and functions including the immune system, cardiovascular system, reproductive success of many species, and reducing the risk of obesity (Connor, 2000; Figueiredo et al., 2017; Fritsche, 2006; Mozaffarian & Wu, 2011; Patterson et al., 2012; Saker et al., 1998). The proper maintenance of fatty acids status can have long-term effects on health, management and conservation of exotic species, especially large herbivores. It has been noted in both rhinoceros and elephant species that managed animals have lower proportions of polyunsaturated fatty acids (PUFAs) and higher omega-6 to omega-3 ratios compared to their free-ranging counterparts (Okuyama, Kobayashi & Watanabe, 1996; Clauss, Grum & Hatt, 2007). In wildlife herbivores these alterations, specifically increased omega-6 to omega-3 fatty acid ratios, is associated with changes to the immune status, skin health, reproductive capabilities, and inflammation (Schmidt et al., 2009; Clauss, Grum & Hatt, 2007). In African elephants, it is well known that atherosclerosis is an issue in managed populations, but it has also been determined that free-ranging elephants on low PUFA diets had an increase in cases of atherosclerosis (Clauss, Grum & Hatt, 2007). Because of the correlation between PUFAs and atherosclerosis in this megaherbivore it can be inferred that diet is an influencer for herbivores.

Currently, there is little information available on the fatty acid status of rhinoceros species, particularly southern white rhinoceroses (Wood et al., 2021c). Black rhinoceros (*Diceros bicornis*) research has found correlations between dietary and circulating omega-6 and omega-3 fatty acids (Clauss et al., 2008). This study and others have found that when dietary omega-6s are higher and omega-3s are lower the same ratios can be seen in circulating fatty acid status of herbivores (Clauss et al., 2008; Grant, Brown & Dierenfeld, 2002; Suedmeyer & Dierenfeld, 1998). This is consistent with other wildlife herbivores as it is well documented that higher grain-based concentrates decrease circulating PUFAs and the omega-6 to omega-3 ratio while higher forage inclusion can increase circulating PUFAs and the omega-6 to omega-3 ratio (Clauss, Grum & Hatt, 2007). Recent studies in southern white rhinoceroses found correlations between dietary differences and circulating fatty acids using DBS cards at two different zoological institutions (Wood et al., 2021c).

While used since the early 1920s in human health monitoring (Freeman et al., 2018), dried blood spot (DBS) filter cards have begun to be used in field research for animals, including fatty acid analysis (Brindle, O’Connor & Garrett, 2014; Dass et al., 2020; Koutsos et al., 2021; Wood et al., 2021a; Wood et al., 2021b; Wood et al., 2021c). This work has been conducted in multiple reptile species (Dass et al., 2020; Koutsos et al., 2021) but validation work to determine if DBS can be directly compared to liquid whole blood in the domestic pig has also been conducted (Wood et al., 2021b). Dried blood spot samples are an important alternative to liquid or frozen whole blood or blood fractions for field work collection because they do not require large amounts of blood to be drawn, only require a few drops of whole blood on specialized filter paper for analysis and potentially have minimal storage needs (no immediate processing or freezing) (Freeman et al., 2018). Additionally, DBS samples are considered safe from biohazards as the drying process inactivates most pathogens (Freeman et al., 2018). With the growth in the use of DBS for animal research it is important to investigate potential limitations of our knowledge regarding this collection method, including the specifics of storage needs for various nutritional biomarkers (Freeman et al., 2018). Most research on fatty acid storage stability in humans focuses on blood fractions, whole blood, blood on chromatography paper that is left wet, and poly-unsaturated fatty acids due to their risk of peroxidation during long-term storage (Halliwell & Chirico, 1993; Metherel & Stark, 2016). Previous studies compared various sample types at room temperature, 4 °C, −20 °C, and −75 °C and found samples stored at −75 °C remained stable for 180 days (Metherel & Stark, 2016). Unfortunately, these studies did not analyze storage stability using DBS samples. Previous studies that have utilized DBS samples, include newborn screening tests to compare temperature storage (−20 °C, 4 °C, ambient or room temperature, and 37 °C) for l-year and longer but these tests do not include fatty acid profiling (Adam et al., 2011; Behets et al., 1992; Cordovado et al., 2009; Fingerhut et al., 2009; Lando et al., 2008; Li et al., 2006; Mei et al., 2001; Strnadova et al., 2007). Due to the notable differences between species, human studies are not the best comparisons for wildlife herbivore species, and it cannot be inferred that DBS samples for fatty acids can be stored the same way as other DBS samples. In addition, research conducted with comparative species is often less funded than human or domestic animal research and therefore it is not uncommon for researchers to store samples for a long time period while waiting for adequate project funding. It is important to know if this additional storage time potential skews research results (Maple, 2008).

The goal of this study was to determine if southern white rhinoceros DBS samples for fatty acid profiling have additional storage limitations by comparing room temperature storage to −80 °C storage and DBS samples sent for analysis immediately. Better understanding the storage parameters of DBS samples could provide full fatty acid profiles with minimal quantities of whole blood and provide important information on the long-term fatty acid status of a variety of species (Armstrong, Metherel & Stark, 2008; Bailey-Hall, Nelson & Ryan, 2008; Baylin et al., 2005).

## Materials and Methods

This study was approved by the NC Zoo Animal Research Committee. Seven adult southern white rhinoceroses (two males and five females) managed at the North Carolina (NC) Zoo, Asheboro, NC, USA were bled *via* medial radial veins in February 2019 during their routine annual exam. Samples were collected in untreated red top vacutainer tubes as whole blood. Animals were trained using positive reinforcement for regular blood draws; thus no restraints were used. Whole blood samples were transferred to Perkin-Elmer Spot Saver cards (Perkin-Elmer, Waltham, MA, USA) with four spots of approximately 80 μL of blood each equating to approximately 320 μL per card. Only one blood spot is needed for the fatty acid analysis, but four spots were collected per animal to provide the opportunity of duplicate analysis in case of any problems during shipping or processing. Each animal had three blood spot cards filled and dried. After drying, one set of dried blood spot cards was shipped within 1 week of sampling to Lipid Technologies (Austin, MN) for a proprietary analysis of a full fatty acid profile including 36 individual fatty acids and 10 fatty acid groups. The second set was stored at room temperature in the NC Zoo veterinary hospital (approximately 23 °C and 20–40% humidity) for 1 year, and the third set was stored in a veterinary clinic ultra-low freezer at −80 °C with approximately 42% humidity for 1 year. The stored samples were kept in a Whirl-Pak® bags (Nasco Sampling/Whirl-Pak®, Madison, WI, USA) and exposed to little or no light as the cards have flaps that cover the spots. Room temperature samples were held in an office desk with limited light and those stored in the ultra-low freezer also had limited light. In February 2020, the two stored sets of samples were sent to Lipid Technologies for the same fatty acid analyses.

During the time period prior to blood collection, the rhinoceroses were provided their normal diets which consists of a bale (approximately 25 kg) of timothy hay (*Phleum pratense*) per animal, access to a 40-acre pasture (fescue (*Festuca arundinacea*), Bermuda (*Cynodon dactylon*), and annual ryegrass (*Lolium multiflorum*)), 1.4 kg Mazuri® Wild Herbivore (St. Louis, MO, USA) pellets daily, and limited supplemental timothy hay cubes for training.

A comparison of the initial samples (*n* = 7), the room temperature storage samples (*n* = 7), and the frozen storage samples (*n* = 7) was conducted using the Kruskal-Wallis *H*-Test with an α ≤ 0.05 and a *H* test statistic of 5.8 to determine if any differences were present among the three DBS sets of fatty acid analyses. If deviations were determined, pairwise comparisons were performed using the Mann-Whitney *U*-Test to determine which groups had differences with an α ≤ 0.05 and a *U* test statistic of 8. All statistical analysis was performed using IBM SPSS Statistics for Windows (Version 25.0 IBM Corp., Armonk, NY, USA). Because of the small sample size and the use of nonparametric statistics, *p*-values are not appropriate. Non-parametric statistics are meant for the analysis of non-normal populations with populations that are smaller than *n* = 20; this low *n*-value lends to higher error values that negate the usefulness of *p*-values unlike traditional parametric statistics thus requiring Kruskal-Wallis *H*-Test and Mann-Whitney *U*-Test to determine differences (Corder & Foreman, 2014).

## Results

Of the fatty acids and groups analyzed, twenty-six of the thirty-six individual fatty acids and 10 fatty acid groups were found in high enough concentrations to be quantified in the collected samples (Table 1). The fatty acids not found in high enough concentrations were lauric acid (12:0), myristoleic acid (14:1), pentadecylic acid (15:0), pentadecanoic acid (15:1), 9-hexadecenoic acid (16:1w5), margaric acid (17:0), heptadecenoic acid (17:1), vaccenic acid (18:1w7), 13-octadecenoic acid (18:1w5), and eicosenoic acid (20:1w9). Pairwise comparisons found all comparisons with statistical differences to be less than a 10% difference between groups and all but one comparison was less than a 5% difference thus it is unlikely that these differences are clinically significant.

**Table 1:** Fatty acid (%) Profile ranges, averages and standard deviations (SD) of initial (*n* = 7), room temperature stored (*n* = 7), and −80 °C stored dried blood spot samples (*n* = 7) from managed Southern White Rhinoceroses (*Ceratotherium simum*) with a Kruskal-Wallis test and pairwise comparisons^1,2,3^.

	Initial		Room temperature (23 °C)		Frozen (−80 °C)	
Individual fatty acid	Range	Average (SD)	Range	Average (SD)	Range	Average (SD)
Myristic acid (14:0)	0.6–1.2	**0.9** ^**x**^ **(0.18)**	0.5–0.8	**0.7 (0.12)**	0.4–1.6	**0.7** ^**y**^ **(0.42)**
Palmitic acid (16:0)	13.6–16.4	**14.9 (1.00)**	14.6–17.2	**16.1 (0.95)**	13.3–16.8	**14.9 (1.19)**
Palmitoleic acid (16:1w7)	1.3–2.4	**1.8 (0.40)**	0.7–2.6	**1.7 (0.68)**	1.2–4.8	**2.2 (1.40)**
Stearic acid (18:0)	11.5–13.4	**12.1** ^**a**^ **(0.66)**	12.5–17.3	**14.3** ^**b**^ **(1.69)**	12.0–15.3	**13.7 (1.31)**
Oleic acid (18:1w9)	32.2–40.1	**35.2 (2.56)**	33.9–40.7	**36.5 (2.22)**	31.5–38.5	**34.2 (2.15)**
Linoleic acid (18:2w6)	22.4–29.9	**26.6** ^**a**^ **(2.89)**	16.8–25.7	**21.7** ^**b**^ **(2.84)**	22.2–25.8	**24.2 (1.29)**
γ-linolenic acid (18:3w6)	0.1–0.8	**0.3 (0.27)**	0.0–0.2	**0.1 (0.06)**	0.0–0.2	**0.1 (0.08)**
α-linolenic acid (18:3w3)	2.1–5.9	**4.2 (1.48)**	1.5–4.9	**3.3 (1.40)**	2.0–5.6	**4.2 (1.46)**
Stearidonic acid (18:4w3)	0.0	**0.0** ^**a,x**^ **(0.00)**	0.2–0.3	**0.3** ^**b**^ **(0.07)**	0.2–1.0	**0.3** ^**y**^ **(0.28)**
Arachdic acid (20:0)	0.0–0.1	**0.1** ^**a,x**^ **(0.04)**	0.3–0.4	**0.3** ^**b**^ **(0.08)**	0.1–0.3	**0.2** ^**y**^ **(0.05)**
Paullinic acid (20:1w7)	0.1–0.2	**0.2** ^**a**^ **(0.07)**	0.6–0.9	**0.6** ^**b**^ **(0.14)**	0.2–0.9	**0.5 (0.21)**
Eicosadienoic acid (20:2w6)	0.3–0.5	**0.4 (0.08)**	0.3–0.7	**0.4 (0.13)**	0.3–0.6	**0.5 (0.13)**
Mead acid (20:3w9)	0.1–0.2	**0.1** ^**a,x**^ **(0.04)**	0.0	**0.0** ^**b**^ **(0.00)**	0.0	**0.0** ^**y**^ **(0.00)**
h-γ-linolenic acid (20:3w6)	0.2–0.4	**0.3 (0.06)**	0.2–0.4	**0.3 (0.06)**	0.2–0.8	**0.4 (0.19)**
Arachidonic acid (20:4w6)	1.2–2.2	**1.8 (0.34)**	1.3–2.0	**1.7** ^**§**^ **(0.26)**	1.4–2.4	**2.1** ^**¤**^ **(0.33)**
Eicosatrienoic acid (20:3w3)	0.1–0.2	**0.1 (0.04)**	0.0–0.2	**0.1 (0.06)**	0.1–0.3	**0.1 (0.06)**
Eicosatetraenoic acid (20:4w3)	0.0–0.1	**0.1** ^**a,x**^ **(0.04)**	0.0–0.4	**0.2** ^**b**^ **(0.11)**	0.2–0.4	**0.3** ^**y**^ **(0.07)**
Eicosapentaenoic acid (20:5w3)	0.1–0.2	**0.1** ^**a**^ **(0.03)**	0.1–0.3	**0.2** ^**b**^ **(0.06)**	0.1–0.4	**0.2 (0.10)**
Behenic acid (22:0)	0.3–0.5	**0.3 (0.08)**	0.3–0.6	**0.4 (0.13)**	0.2–0.4	**0.3 (0.06)**
Erucic acid (22:1w9)	0.0	**0.0** ^**x**^ **(0.0)**	0.0–0.1	**0.0 (0.04)**	0.0–0.2	**0.1** ^**y**^ **(0.06)**
Docosatetraenoic acid (22:4w6)	0.0–0.1	**0.0** ^**a,x**^ **(0.05)**	0.1–0.3	**0.2** ^**b**^ **(0.04)**	0.1–0.2	**0.2** ^**y**^ **(0.03)**
DPA (osbond) (22:5w6)	0.0–0.3	**0.1 (0.11)**	0.1–0.3	**0.2 (0.05)**	0.1–0.2	**0.1 (0.04)**
DPA (clupanodonic) (22:5w3)	0.0–0.2	**0.1 (0.05)**	0.0–0.2	**0.1** ^**§**^ **(0.04)**	0.1–0.2	**0.2** ^**¤**^ **(0.03)**
DHA (22:6w3)	0.1–0.2	**0.2 (0.04)**	0.1–0.3	**0.2 (0.07)**	0.1–0.2	**0.1 (0.04)**
Lignoceric acid (24:0)	0.3–0.5	**0.4** ^**a,x**^ **(0.06)**	0.0	**0.0** ^**b**^ **(0.00)**	0.0	**0.0** ^**y**^ **(0.00)**
Nervonic acid (24:1)	0.1–0.3	**0.2** ^**a,x**^ **(0.07)**	0.3–0.5	**0.4** ^**b**^ **(0.07)**	0.2–0.4	**0.3** ^**y**^ **(0.06)**
**Fatty acid group**	**Range**	**Average (SD)**	**Range**	**Average (SD)**	**Range**	**Average (SD)**
Saturates	26.4–29.5	**28.3** ^**a**^ **(0.99)**	29.0–33.3	**31.9** ^**b**^ **(1.56)**	26.7–31.6	**29.9 (1.68)**
Monoenes	32.6–40.7	**35.6** ^**a**^ **(2.64)**	34.9–41.8	**37.5** ^**b**^ **(2.28)**	32.4–35.7	**35.1 (2.02)**
Poly Unsaturated Fatty Acids (PUFA)	29.0–39.1	**34.3** ^**a**^ **(3.07)**	23.0–34.9	**29.0** ^**b**^ **(3.55)**	31.9–34.2	**32.8 (1.02)**
Highly Unsaturated Fatty Acids (HUFA)	2.3–3.4	**2.9** ^**x**^ **(0.39)**	2.7–3.7	**3.2 (0.39)**	3.4–3.9	**3.6** ^**y**^ **(0.19)**
Total w3	2.8–6.6	**4.8 (1.53)**	2.2–6.0	**4.7 (1.55)**	3.0–6.9	**5.4 (1.62)**
Total w6	24.4–33.2	**29.4** ^**a**^ **(3.23)**	19.1–28.9	**24.6** ^**b**^ **(3.11)**	25.1–29.3	**27.4 (1.53)**
Total w9	32.4–40.6	**35.4 (2.67)**	34.3–41.2	**36.9 (2.29)**	32.0–38.7	**34.6 (2.10)**
w6/w3	4.8–12.0	**7.0 (3.30)**	4.4–11.3	**6.5 (3.26)**	3.6–9.9	**5.8 (2.56)**
Omega 3 HUFA	15.1–28.0	**21.3 (3.90)**	15.9–32.2	**25.6 (5.18)**	21.9–28.7	**24.0 (2.29)**
Omega 6 HUFA	72.0–84.9	**78.7 (3.90)**	67.8–84.1	**74.4 (5.18)**	71.4–78.1	**76.1 (2.29)**

**Notes:**

^1^Differing superscripts (^x,y^) in averages columns are significantly different at (α = 0.05) for initial compared to −80 °C.

^2^Differing superscripts (^a,b^) in averages columns are significantly different at (α = 0.05) for initial compared to room temperature.

^3^Differing superscripts (^§,¤^) in averages columns are significantly different at (α = 0.05) for room temperature compared to −80 °C.

Pairwise comparisons for initial DBS samples with immediate analysis and −80 °C frozen 1-year stored samples found eight fatty acids and one fatty acid group with differences (Table 1). Of those, four fatty acids had higher concentrations in the frozen samples compared to the initial samples and four fatty acids and one fatty acid group had higher concentrations in the initial samples than in the frozen samples. The fatty acids that had higher concentrations in the frozen samples were stearidonic acid (18:4w3) (Δ 0.3%), arachdic acid (20:0) (Δ 0.1%), eicosatetraenoic acid (20:4w3) (ETA) (Δ 0.2%), and erucic acid (22:1w9) (Δ 0.1%). The fatty acids that were higher in the initial samples were myristic acid (14:0) (Δ 0.2%), mead acid (20:3w9) (Δ 0.1%), docosatetraenoic acid (22:4w6) (DTA) (Δ 0.2%), nervonic acid (24:1) (Δ 0.1%), and total highly unsaturated fatty acids (HUFAs) (Δ 0.7%).

Pairwise comparisons for initial DBS samples with immediate analysis and 1-year room temperature stored samples found 10 individual fatty acids and five fatty acid groups with differences (Table 1). Of those with differences, eight fatty acids and two groups had concentrations that were higher in the 1-year stored room temperature samples than the initial samples including: stearic acid (18:0) (Δ 2.2%), stearidonic acid (18:4w3) (Δ 0.3%), arachdic acid (20:0) (Δ 0.2%), paullinic acid (20:1w7) (Δ 0.4%), ETA (20:4w3) (Δ 0.1%), eicosapentaenoic acid (20:5w3) (EPA) (Δ 0.1%), DTA (22:4w6) (Δ 0.2%), nervonic acid (24:1) (Δ 0.2%), monoenes (Δ 1.9%), and total saturates (Δ 3.6%). The other two fatty acids and three fatty acid groups were higher in the initial samples than in the 1-year stored room temperature samples included linoleic acid (18:2w6) (Δ 4.9%), mead acid (20:3w9) (Δ 0.1%), total polyunsaturated fatty acids (PUFAs) (5.3%), and total omega-6 fatty acids (Δ 4.8%).

Pairwise comparisons for room temperature storage and −80 °C frozen storage samples had only two fatty acids with differences, arachidonic acid (20:4w6) (Δ 0.4%) and omega-3 docosapentaenoic acid (DPA) (22:5w3) (Δ 0.1%), which were both higher in the frozen samples than in the room temperature samples (Table 1).

## Discussion

The method of storage had no significant effects on the essential fatty acids (EFAs) linoleic acid (18:2w6) and α-linolenic acid (18:3w3). Similarly, their downstream partners eicosapentaenoic acid (EPA) and docosahexaenoic acid (DHA) and the important omega-3 and omega-6 fatty acid groups and PUFAs were minimally altered *via* storage method overall. Of these critical values, only linoleic acid, total omega-6 fatty acids, and PUFAs displayed higher concentrations in initial samples than room temperature samples. This is consistent with values found in domestic pigs when non-statistically comparing fatty acid percentages found in a liquid whole blood sample to a DBS sample sent immediately for analysis, a DBS sample stored at room temperature for 1 year, and a DBS sample stored at room temperature for 2 years (Wood et al., 2021b). This study confirmed that, unlike other biomarkers stored as DBS samples, fatty acids are impacted by temperature and storage time due to oxidation. Metherel, Aristizabal Henao & Stark (2013) found that human samples collected on chromatography paper kept at room temperature had a maximum stability period of 30 days before PUFAs began to degrade. Chromatography paper is similar to DBS cards but has less stability than the DBS cards because it lacks the specialized filter paper used in DBS cards (Brindle, O’Connor & Garrett, 2014). The deterioration of fatty acids seen in chromatography paper was also seen in room temperature DBS samples collected in this study. This coupled with the lack of significant changes in total omega-3 or the omega-6 to omega-3 ratio, is positive for DBS being a useful handling and storage method in field research especially when there is not frozen storage available for short term storage.

There were only two fatty acids, arachidonic acid (20:4w6) and omega-3 DPA (22:5w3) with differences when comparing the stored frozen samples to the stored room temperature samples, and both fatty acids were higher in the frozen samples. For field work, this suggests that samples can be kept at an ambient temperature for a short period in the field while being transported to labs or frozen storage but freezing samples that will be stored long term is recommended. It also suggests that further research with shorter intervals for longer than 1 year of storage at both temperatures (*i.e*., 1 month, 3 months, 6 months, etc.) to determine the “limit” is warranted. Determining the true limit of these samples is critical for wildlife research as funding may not always be readily available for both collection and analysis of samples at the same time (Maple, 2008). Additionally, understanding the “limit” for room temperature storage can provide field researchers with more information to use to avoid extreme losses in samples when frozen storage may not be readily available.

The more significant storage method differences were seen when comparing the initial samples to the two types of storage methods, implying that storage does impact the stability of fatty acids on DBS cards. Of the two commonly used storage methods for DBS cards, this data resulted in fewer differences between the initial and frozen samples which supports the theory that frozen samples are more stable, but the differences found may only be statistically significant, not clinically significant. Focusing on the individual fatty acids, 15 of 36 had statistically significant differences among three storage methods, equating to 42% of the total individual fatty acids found. It is important to note that twelve of these individual fatty acids, or 33% of the total individual fatty acids found and 80% of the fatty acids with statistical differences, are non-essential and trace fatty acids with very low percentage concentrations thus the differences noted may not be clinically significant or relevant for these animals.

Even if the differences are not clinically significant, further detailed research comparing larger populations for statistical strength and/or shorted periods of frozen storage could assist in understanding how long samples can remain stable. Additionally, four of the nine fatty acids with differences resulted in increases with storage time at -80 °C. This could be due to minor analytical differences between the two times the samples were measured or desiccation in the freezer. Some of these statistical variations could also be due to the small sample size and the variations among the individual animals.

Due to the results of the current research fatty acid samples, even with frozen storage, we recommend that when samples are collected, they are analyzed as quickly as feasible and that they do not remain in storage for longer than 1 year. Laboratories that analyze these samples need to prioritize these analyses so that they do not delay the analysis further. If a study is comparing multiple populations or species, it is preferred that storage and analysis time be kept as consistent as possible and samples be analyzed using the same laboratory and analytical runs or same techniques verified between laboratories.

Of the fatty acids with differences between the initial samples and the frozen samples, those with clinical importance to white rhinoceroses included stearidonic acid and mead acid. Stearidonic acid is a precursor for omega-3 fatty acids and is a metabolite of α-linolenic acid which can be converted into DHA and EPA (Guil-Guerrero, 2007). This fatty acid has been found to have a positive impact on the efficiency of conversion to EPA and later DHA in humans when provided in the diet and is critical for brain function and spermatozoa health (Gerster, 1998). In African savanna elephants (*Loxodonta africana*), α-linolenic acid is critical for the reproductive health of males, specifically their spermatozoa development (Dierenfeld, 2006). It can be inferred that this α-linolenic acid importance is also due to the downstream conversion to DHA. For southern white rhinoceroses, α-linolenic acid and stearidonic acid could play an important role in reproductive health and success. Because it has been determined that, like other herbivorous wildlife, fatty acids are influenced by the diet of southern white rhinoceroses (Wood et al., 2021c) it is important to understand how these circulating values can be used for dietary changes. The results of this study found that stearidonic acid was statistically higher in both temperature storage sample sets when compared to the initial samples and thus storage could alter published normal values.

There is some discussion on whether humidity is important to the stability of DBS samples. While some studies have found that lower humidity levels (below 30%) had better sample stability than higher humidity (above 50%) (Adam et al., 2011) other studies found humidity of sampling, drying, and storage location had no impact on sample stability (Denniff, Woodford & Spooner, 2013). Unlike the previous human studies, the current study did not monitor or control humidity levels within the two temperature storage conditions. The room temperature samples were kept in a temperature-controlled office within the NC Zoo veterinary hospital under average humidity (20–40%) conditions. While the low humidity inside the −80 °C freezer (housed in the same veterinary hospital) would be preferable, the variable humidity levels could be the reason why there were more differences between the initial samples and the room temperature samples. Further studies in DBS storage protocols for animal fatty acids should include humidity and temperature monitoring to better correlate variables.

While whole blood can offer better information on long-term fatty acid status of an animal, it also has the caveat of fatty acid results being influenced by the levels of white blood cells and hematocrit within the sample, thus potentially leading to higher levels of fatty acids that may be difficult to interpret (Brenna et al., 2018). Despite these concerns, the temperature data collected here does support previous biochemical marker studies in humans that determined that at least -20 °C provided longer analyte stability in storage (1-year or longer) (Adam et al., 2011; Behets et al., 1992; Cordovado et al., 2009; Fingerhut et al., 2009; Lando et al., 2008; Mei et al., 2001; Strnadova et al., 2007). While these prior studies are helpful in validating the current findings, none of them directly analyzed fatty acids but rather analyzed disorder/disease markers, small metabolites, and amino acids. Additionally, work performed in African savanna elephants comparing DBS samples to liquid whole blood, serum, and plasma found minimal differences between the four sample types, suggesting that comparing across sample type is possible (Wood et al., 2021a). A study conducted by Nurhasan et al. (2015) focused on fatty acid stability of whole blood in humans with varying storage temperatures that included comparing −20 °C to −80 °C storage (9–11 months). The results of this study found that there were more highly unsaturated fatty acids and a lower omega-6 to omega-3 ratio in samples stored at −20 °C (Nurhasan et al., 2015). While valuable, other research suggests that −75 °C or below is better for PUFA and total lipid extracts in serum, plasma, whole blood, and chromatography paper as it stops nearly all peroxidation (Metherel & Stark, 2016). Outside of monitoring humidity and including more time points for comparison, these differing results suggest that a comparison of −20 °C and −80 °C storage is warranted for DBS samples to determine which temperature is better suited to this method of blood collection or if individual fatty acids or fatty acid groups may have different optimal storage methods.

## Conclusions

Data from this study found only two individual fatty acid differences between the two storage types when comparing samples held for 1 year, but there were more differences when comparing initial samples to frozen and room temperature storage samples. It was found that the frozen samples had the fewest statistical differences and support previous findings of being the most advantageous form of storage for DBS samples used to study fatty acids. When looking at the important omega-3 and -6 fatty acids, differences were not found between the initial and frozen samples, lending to the use of frozen storage for analysis of these key fatty acid groups. More specifically, linoleic acid and PUFAs are most stable if sent for analysis immediately, but storage at -80 °C had minimal impact on stability and is recommended for these fatty acids. If the interest lies in HUFA analysis, immediate analysis is recommended as storage at -80 °C had statistically significant degradation after 1 year. The lack of differences also displays that DBS can be used as a simple and effective collection method to monitor fatty acid status in southern white rhinoceroses, although further research is needed to determine if shorter term DBS fatty acid storage is most recommended, especially in EFAs such as EPA.

## Supplemental Information

10.7717/peerj.12896/supp-1 Supplemental Information 1 Fatty Acid (%) Profile of Initial Dried Blood Spot (DBS) Samples (*n* = 7), Room Temperature Stored DBS Samples (*n* = 7), and −20 °C Stored DBS Samples from Human Managed Southern White Rhinoceroses (*Ceratotherium simum*). The values for seven rhinos with DBS samples collected and either immediately sent for analysis or stored at two different temperatures. These values were used for statistical comparisons of three sample groups.

## Additional Information and Declarations

### Data Availability

The following information was supplied regarding data availability: The raw data are available in the Supplemental File.
